# The Link Between Sex Hormones and Susceptibility to Cardiac Arrhythmias: From Molecular Basis to Clinical Implications

**DOI:** 10.3389/fcvm.2021.644279

**Published:** 2021-02-17

**Authors:** Sarah Costa, Ardan M. Saguner, Alessio Gasperetti, Deniz Akdis, Corinna Brunckhorst, Firat Duru

**Affiliations:** ^1^Arrhythmia and Electrophysiology, Department of Cardiology, University Heart Center, Zurich, Switzerland; ^2^Cardiac Arrhythmia Service, Department of Cardiology, Johns Hopkins Hospital, Baltimore, MD, United States; ^3^Center for Integrative Human Physiology, University of Zurich, Zurich, Switzerland

**Keywords:** arrhythmia, sex hormones, cardiomyopathy, channelopathy, testosterone, estrogen

## Abstract

It is well-known that gender is an independent risk factor for some types of cardiac arrhythmias. For example, males have a greater prevalence of atrial fibrillation and the Brugada Syndrome. In contrast, females are at increased risk for the Long QT Syndrome. However, the underlying mechanisms of these gender differences have not been fully identified. Recently, there has been accumulating evidence indicating that sex hormones may have a significant impact on the cardiac rhythm. In this review, we describe in-depth the molecular interactions between sex hormones and the cardiac ion channels, as well as the clinical implications of these interactions on the cardiac conduction system, in order to understand the link between these hormones and the susceptibility to arrhythmias.

## Introduction

Cardiac arrhythmias encompass a wide spectrum of clinical presentations, ranging from benign extrasystoles on electrocardiogram (ECG) to arrhythmias that may pose a significant clinical threat, such as atrial fibrillation (AF), which is an important cause of stroke, and ventricular tachycardia or fibrillation (VT/VF) that can result in sudden cardiac death (SCD) (1). A significant proportion of SCD events occurs in patients without any known cardiac disease, and even in the presence of diagnosis of a cardiac disorder, current knowledge on prediction of arrhythmic risk is limited (2). It is well-known that gender is an independent risk factor for some types of cardiac arrhythmias. For example, males have a greater prevalence of AF and the Brugada Syndrome (3, 4). In contrast, females are at increased risk for Torsade de pointes (TdP), both in congenital and acquired Long QT Syndrome (LQTS) (5). Despite the fact that the incidence of a variety of cardiac arrhythmias differs between men and women, the underlying mechanisms of these gender differences have not been clearly identified.

The predominant factors determining gender differences are the sex hormones. These steroid hormones are known to exert their multiple physiological effects by binding to cytosolic or membrane receptors (6). One of the target organs of action of these hormones is the heart, and there is now accumulating evidence indicating that sex hormones may have a significant impact on the cardiac rhythm (7, 8). It was initially thought that sex hormones only had genomic actions, such as exerting their effects through the regulation of transcriptional processes, hence influencing gene expression after nuclear translocation (9). However, recent literature has shown that their scope of action goes well-beyond that, and encompasses non-genomic actions, as well. In fact, these hormones can acutely regulate cardiac ion channels and affect their currents, and these actions are mediated by transcriptional processes, such as RNA and protein synthesis (6–8).

In this review, we aim to describe in-depth the molecular interactions between sex hormones and the cardiac ion channels, as well as the clinical implications of these interactions on the cardiac conduction system, in order to understand the link between these hormones and the susceptibility to arrhythmias.

## Types and Functions of Sex Hormones

**Figure 1:**
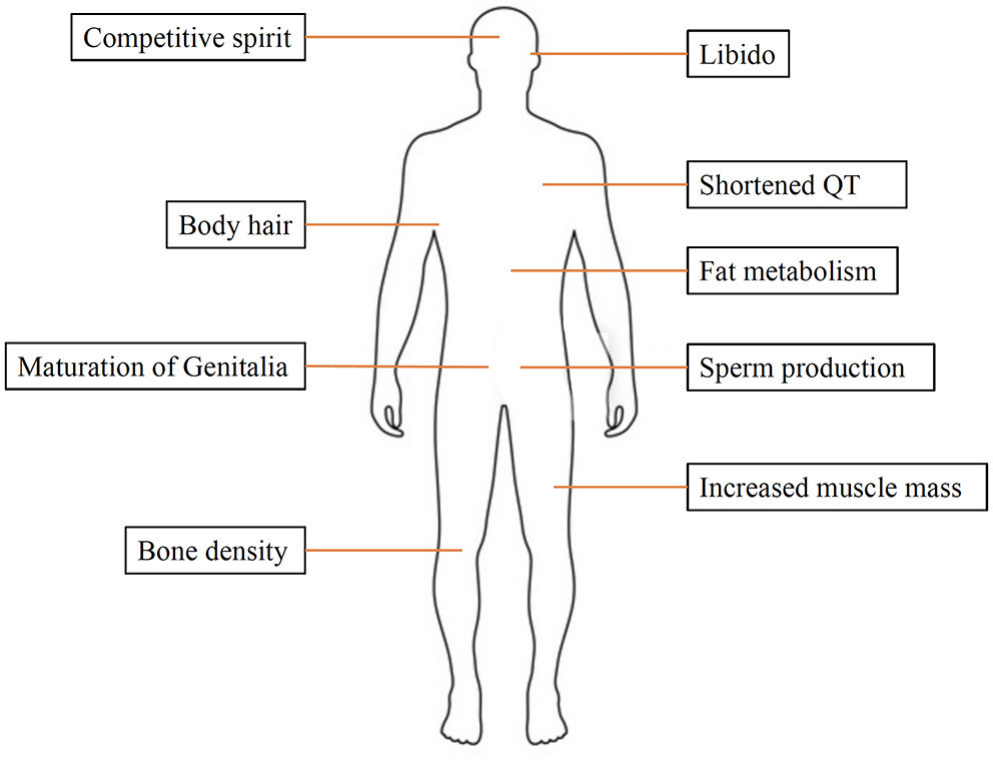
The effects of testosterone on the human body.

Sex hormones play a key role in reproduction and sexual development. Moreover, they are also involved in other processes, such as regulating cholesterol levels and determining inflammatory response. They are produced by the gonads (ovaries and testicles) and adrenal glands. Estrogen and progesterone are the two main female sex hormones. Estrogen promotes growth of uterine tissue and breasts, maintains libido and secondary sexual characteristics in females, whereas progesterone has a significant role in the female menstruation cycle and pregnancy. The main male sex hormone is testosterone, but females also produce a small amount of this hormone. Testosterone affects many organs and has a variety of functions, including spermatogenesis, muscle growth, maturation of genitalia and bone metabolism (Figure 1).

### Fluctuations in Sex Hormone Levels

The levels of sex hormones change during life as part of physiological processes. For example, the female sex hormones increase during adolescence and drop during menopause. Moreover, cyclic hormonal changes occur during the reproductive cycle. In certain situations, fluctuations of sex hormone levels can lead to health issues such as infertility, change in sexual desire, hair loss, osteoporosis, etc.

Sex hormone fluctuations also occur in the short-term. For example, there is a substantial circadian variation in the circulating levels of testosterone. Normal values for testosterone in a healthy adult male range between 300 and 1,000 ng/dL (10). The highest level of testosterone is reached in the morning, and therefore, it is recommended to determine it between 8:00 and 10:00 am in order to achieve accurate (and comparable) measurements (11). In addition, there may also be a seasonal variability of testosterone measurements. However, this is a contradictive issue, since there is both literature supporting and rejecting this hypothesis (12). After the third decade of life, men start to have ~1–2% decrease of testosterone per year (13). Finally, the amount of active serum testosterone is heavily reliant on the concentrations of sex hormone-binding globulin (SHBG) and albumin, thus being subject to fluctuations in those proteins. Such changes of testosterone levels across the healthy population pose challenges in determining its effect on cardiac physiology, as values measured have to be adjusted for multiple factors, in order to have an unbiased result.

**Figure 2:**
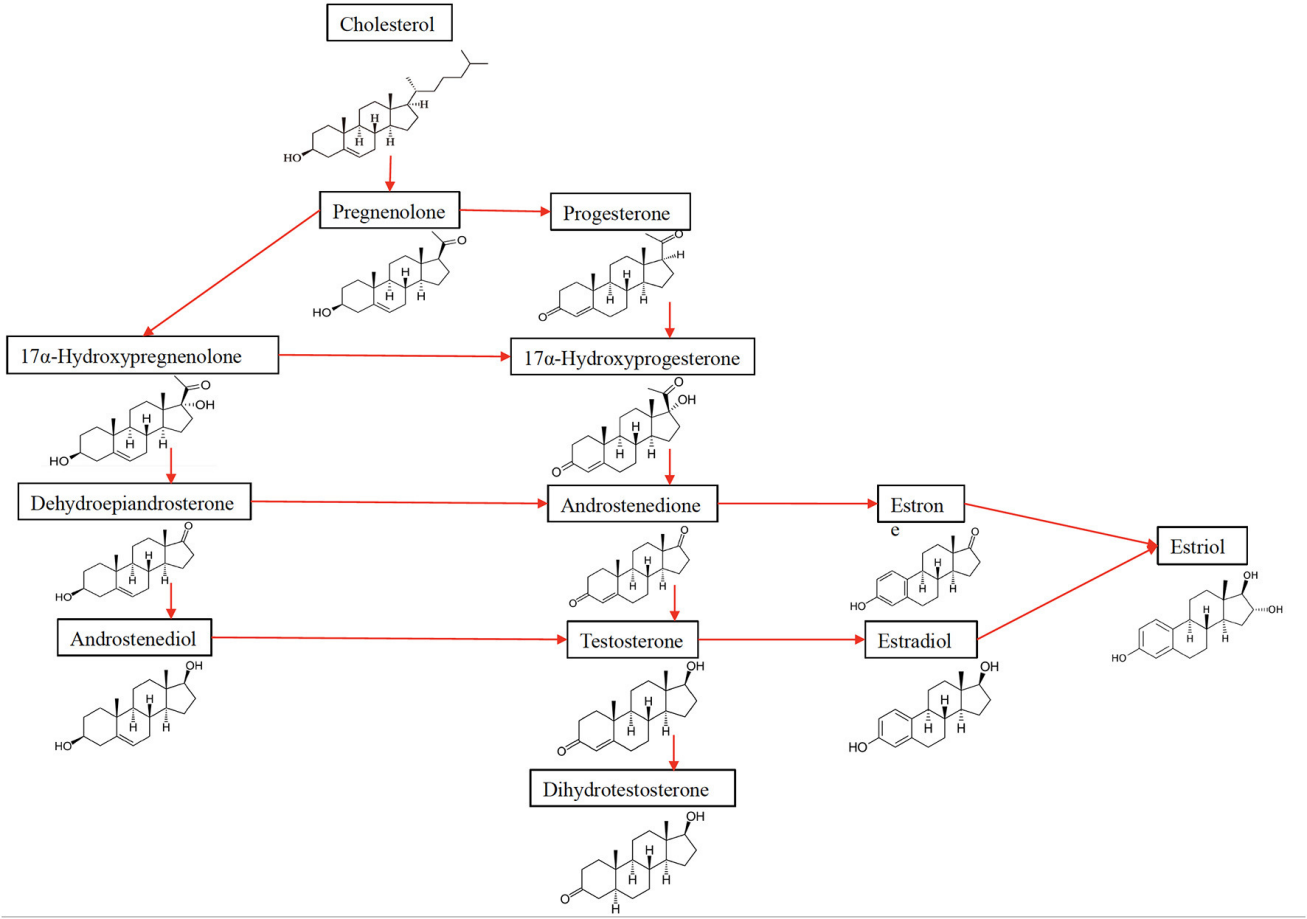
The synthesis of sex hormones from their precursor cholesterol.

Estradiol (E2), the most potent estrogen, has the function of regulating reproductive cycles in females (Table 1, Figure 2). Evaluating estradiol levels is challenging, given that it changes widely during the menstrual cycle. Estradiol increases prior to ovulation and falls during ovulation, and there is a gradual increase of estradiol and then decrease prior to its lowest point in early menses. Moreover, it is also subject to a circadian variability, which is further affected by the menstrual cycle in post-menopausal women, estrogen levels decrease and show fluctuations (14).

**Table 1:** Sex hormones and their biological functions.

**Sex hormones**	**Biological functions**
**Androgens**	
Testosterone	Development of male genitalia, increase in muscle and bone mass, growth of body hair
Dihydrotestosterone	Catalyzed from testosterone, considerably more potent agonist of the androgen receptor
Androstenedione	Precursor of testosterone, endogenous pro-hormone, weak androgenic, and estrogenic activity
**Estrogens**	
Estrone (E1)	Weak estrogen, precursor of estradiol
Estradiol (E2)	Potent estrogenic hormone: regulation of the estrous and menstrual female reproductive cycle, development of female genitalia, bone growth and density, skin health, neuroprotective
Estriol (E3)	Weak estrogen, almost undetectable in non-pregnant women, high levels during pregnancy
Estetrol (E4)	Weak estrogen found only in pregnancy, physiological function unknown

### Sex Hormones and Exercise

Both testosterone and estrogen play an important role in the neuromuscular adaptation to exercise in males and females. This adaptation is mediated by the hypothalamic-pituitary-gonadal (HPG) axis, which is responsible for both acute and chronic responses to exercise (15). In males, testosterone is pivotal for athletic activity during adolescence and in adulthood. While it is well-known that exercise acutely increases testosterone levels in men, its impact in the long-term is much less clear (16). Multiple cross-sectional studies have identified decreased testosterone levels in endurance and resistance athletes, as compared to controls. Furthermore, a study by Grandys et al. (17) found that testosterone levels vary significantly even within the athlete group, depending on the training period. In fact, testosterone increases during low-intensity training periods in comparison to high-intensity training periods. Conversely, in a cross-sectional study by Fitzgerald et al. (18) which compared trained cyclists to recreational cyclists, the former group had higher levels of serum testosterone than the latter. The conflicting results could be due to the retrospective nature of the studies evaluating the impact of chronic exercise. On the other hand, the few prospective studies also showed contradictory results, most likely due to differences in the training period, the magnitude of training stimulus and the volume of training load employed. In females, the acute effects of exercise training on the HPG axis are less well-known. Nindl et al. (19) have shown that estradiol may increase acutely after exercise, whereas the long-term effects of exercise still need to be determined.

## The Interaction Between Sex Hormones and Cardiac Ion Channels

The differences in cardiac electrical activity between males and females had been observed with the earliest ECG recordings a century ago. In the meantime, while the scientific community has gathered a thorough understanding of the ECG as the net sum of electrical current flow of ions crossing the cardiomyocyte cell membrane, the molecular basis underlying gender differences are still largely debated (20).

The transit of ions across the cell membrane is permitted by specific transmembrane proteins, i.e., ion channels. Any change that affects the flow of ions through these channels will have an impact on the ECG (21). The voltage-gated channels are the major ion channel group in charge of cardiac electric activity. These possess a rapid response mechanism to changes in membrane voltage and interact with each other to produce the cellular action potential (AP). In the myocardium, the rapidly activating sodium channel is the principal driver for cellular depolarization, while L-type calcium current is responsible for the plateau phase and the delayed rectifier potassium current is mostly in charge of the repolarization. These give rise to the clinically measurable QRS complex, the magnitude and dispersion of which are strongly influenced by changes to the sodium current, and the QT interval, which is dependent on the calcium and potassium currents (22). These parameters are also widely influenced by cell-cell connections, which are mediated by the connexin family of proteins at gap junctions, and therefore, ECG abnormalities often occur in diseases of the connexome (e.g., arrhythmogenic right ventricular cardiomyopathy). A decrease in cell-cell conductance typically leads to a longer QRS duration with or without terminal activation delay (23). Since the above-mentioned gender differences are not present at birth, but only appear at puberty, this has led to the hypothesis that they are dependent on sex hormone regulation (24).

**Figure 3:**
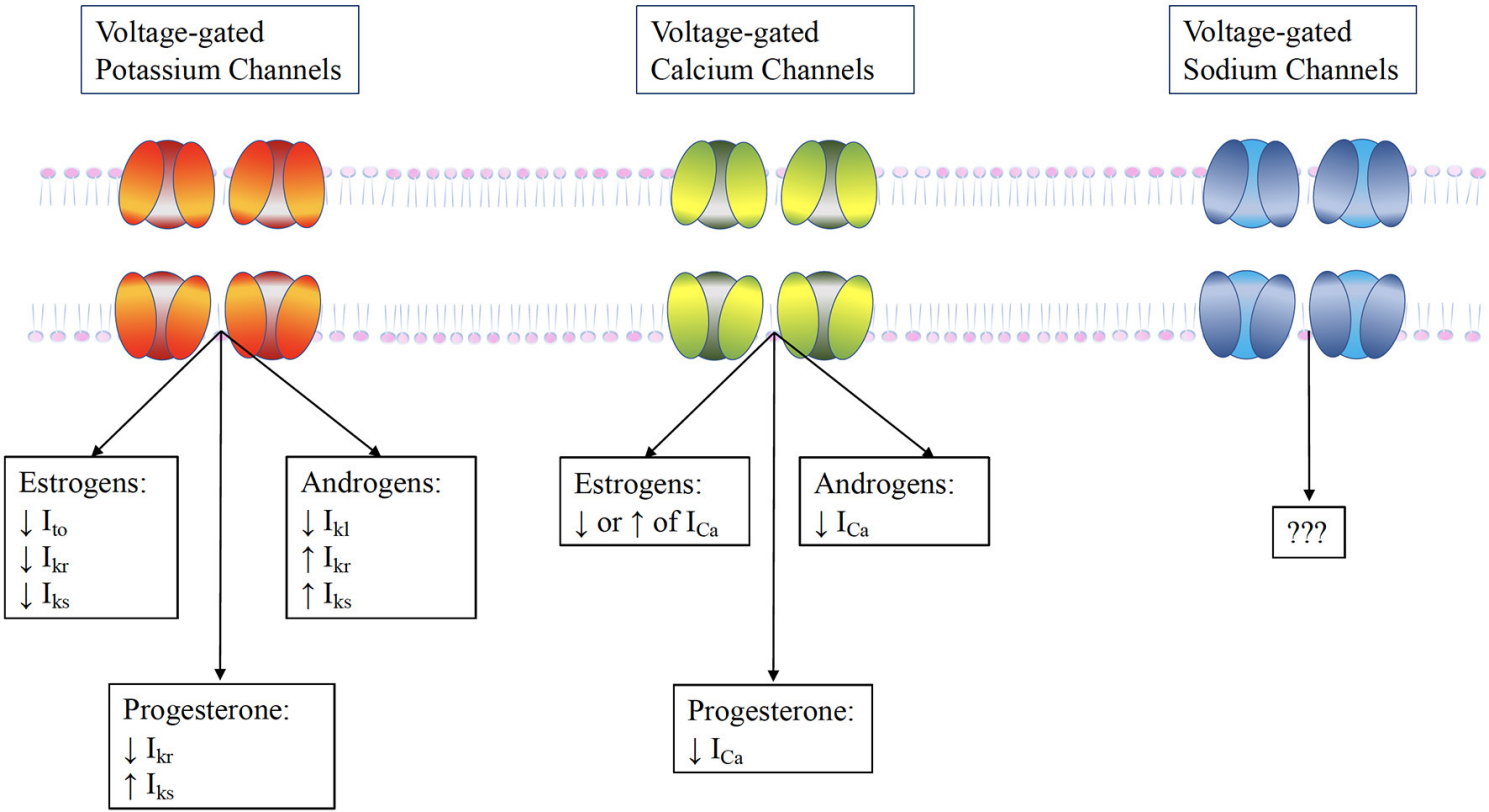
The influence of sex hormones on ventricular ion channels. Testosterone increases repolarizing currents I_kr_ and I_ks_ and the I_to_, while it acutely increases the L-type calcium current I_CaL_. Estrogen has a more complex effect. It directly blocks the I_kr_ , but may also increase it by promoting HERG trafficking. Furthermore, it reduces the KCNE1, thereby reducing I_ks_. These alterations result in prolongation of AP duration by estrogens, but its shortening by testosterone.

Cardiac myocytes have a variety of receptors for sex hormones, specifically estrogen, progesterone and testosterone, whose activation can alter the electrical activity of the heart through modulation of ion channels. Although literature is scarce, fluctuating hormone levels can lead to changes in the behavior and expression of myocardial ion channels (Figure 3) (25).

### The Effect of Sex Hormones on Ventricular Ion Channels

#### Ventricular Potassium Channels

Potassium channels play a pivotal role in the regulation of muscle excitability. Potassium conductance through potassium channels present on the plasma membrane enables the membrane potential to reach the equilibrium potential of potassium, leading to hyperpolarization or accelerated repolarization of action potential in muscle cells. This results in a controlled cell excitability, elicited by various signals such as depolarization of membrane potential, ligand-binding, and so on (26). Sex hormones can regulate these via modulation of gene expression (by binding to their unique nuclear receptor) or non-genomic pathways after binding to membrane receptors. The rapid non-genomic effects of sex hormones are exerted through the activation of specific signaling pathways, which are initiated from sex hormone receptors or binding proteins in the plasma membrane and cytosol. Furthermore, sex hormones may also directly bind to potassium channels or the auxiliary subunits to modulate the activities of potassium channel blockers and potassium channel openers.

Several different types of currents pass through the potassium channels, i.e., the transient outward current (I_to_), the rapid-delayed rectifier current (I_kr_), the slow delayed-rectifier current (I_ks_), and the inward rectifier potassium current (I_kl_). However, there are contradicting reports concerning the interaction of sex hormones and the potassium channels. Previous studies either showed no influence of sex hormones on I_to_ (27) or greater expression in males than in females (28, 29). Consequently, an increased expression of this current was demonstrated in ovariectomized mice, with a concurrent increase in their associated channel mRNA (30). This has been confirmed by the fact that in the high estradiol state (as is pregnancy), a reduction of I_to_ is observed (31).

Similarly to the I_to_, the I_kr_ has also been shown to be either not influenced by gender (29), or less expressed in females (32). This was confirmed in an *in vitro* study upon acute administration of exogenous estradiol on cultured guinea pig cardiomyocytes with human ether-a-go-go-related gene (hERG) overexpression, which unmasked the blockage of the hERG channel and thus I_kr_ (33). In various animal models, the I_ks_ has been observed as either larger in males (34), larger in females or with no gender difference at all (29), depending on which region the cells were taken from. This is in line with increasing evidence suggesting that the differences exerted by sex hormones may not be uniform across the heart. As in the case of I_kr_, this current has also been shown to be blocked upon administration of exogenous estradiol through blockage of its related channels (KCNQ1/KCNE1) (35). Interestingly, while this current is enhanced by progesterone, as shown in the *in vitro* patch clamping study by Nakamura et al. (36) the hERG channel trafficking is reduced with progesterone. Furthermore, it has been demonstrated in guinea pig ventricular myocytes through acute applications of physiological serum levels of testosterone that this increases the outward potassium currents (I_kr_ and I_ks_) and the inward rectifier current (I_kl_) (7). Thus, progesterone and testosterone shorten the ventricular APs, while estrogen lengthens the APs and may exert a pro-arrhythmic effect (8).

#### Ventricular Calcium Channels

The L-type Ca^2+^ channels are heteromultimeric proteins, consisting of multiple subunits. Similar to the mechanism with the above-mentioned potassium channels, signaling of gonadal steroids has traditionally been associated to a genomic action, i.e., the transcriptional control of target genes through binding of the nuclear receptors and ligands to the genomic consensus sequence in reproductive organs. However, several biological actions of gonadal steroids have recently been shown to be too rapid to be compatible with transcriptional mechanisms.

The evidence on interaction of sex hormones and the cardiac calcium channels is conflicting. In fact, there are reports of increased (29), decreased (32), or indifferent calcium currents (I_Ca_) in females as compared to males (27). Similarly to what was observed for the cardiac potassium channels, there is evidence of regional heterogeneity in the different interactions between sex hormones and ion channels (37). In fact, in animal studies, gender differences were observed for the I_Ca_ in the apex, but not in the base of the left ventricle of rabbits (37). Moreover, an increase in L-type calcium channels was shown in the basal cardiomyocytes of rabbits by incubating these with physiological concentrations of estrogen. This effect, however, was not seen in the apical cardiomyocytes (38). Acute administration of progesterone was shown to decrease the I_Ca_ by 60% (8). Interestingly, ovariectomized mice had an increase in the expression of L-type calcium channels, which were reversed upon administration of exogenous estrogen (39). Testosterone also plays a part in the modulation of the voltage-gated calcium channels. It is a selective and potent inhibitor of L-type calcium channels of vascular smooth muscles (40). The likely reason may be the fact that it blocks the major α_1_ subunit of the L-type channel, similarly to the effects of the commonly prescribed calcium channel antagonists.

#### Ventricular Sodium Channels

The influence of sex hormones on the cardiac sodium channels are yet unknown. Even though there is no reported association between sex hormones and the LQT3 (41), there is increasing evidence linking sex hormones to the phenotypic expression of the Brugada Syndrome (42), despite the fact that both diseases stem from cardiac sodium channel mutations. The interesting link between the Brugada Syndrome and sex hormones is thoroughly discussed in the later section.

#### Ventricular Ryanodine Receptor

The contractile function of the heart is determined by intracellular calcium concentration, which is influenced by the expression and activity of calcium regulatory proteins. When the sarcolemma is depolarized, the voltage-gated L-type calcium channels are opened and the influx of calcium into cytosol increases. This influx induces a massive release of calcium from the sarcoplasmic reticulum via the ryanodine receptor (RyR). This results in a sudden increase in calcium, which then triggers cardiac contraction. Tsang et al. (43) demonstrated how testosterone increased contraction and relaxation velocities that were associated with increased calcium release and recovery through activities of the RyR receptor and sarcoendoplasmic reticulum calcium transport ATPase (SERCA2). This observation may explain the predominantly male phenotype in catecholaminergic polymorphic ventricular tachycardia (CPVT).

## Sex Hormones and Ventricular Arrhythmias

Sex hormones may alter the susceptibility to ventricular tachyarrhythmias in patients with underlying structural heart disease and in those with cardiac channelopathies.

### Coronary Artery Disease

The higher incidence of coronary artery disease, and especially of SCD, in male patients but also in females following menopause, has highlighted the role of estrogen as a cardioprotective hormone. It was shown that estrogen has beneficial effects by improving cardiac function, preserving calcium homeostasis and inhibiting the mitochondrial apoptotic pathway (44). In the context of an ischemic insult to the heart, reperfusion that accompanies the opening of a blocked coronary artery may trigger arrhythmias and result in SCD. Estrogen was shown to decrease reperfusion arrhythmias in multiple animal studies. Savergnini et al. (45) demonstrated in young female rats that administration of estradiol was protective against this type of arrhythmia. This finding was also confirmed by Wang et al. (46) who showed that this action was mediated by the genomic action of estrogen, mainly the upregulation of the Estrogen Receptor β (Erβ) activation.

The role of testosterone during cardiac ischemia and in the prevention of reperfusion arrhythmias is controversial. Preclinical studies showed that pretreatment with testosterone of rat hearts exposed to ischemia decreased arrhythmias, as effectively as it was the case after estradiol administration (47). Likewise, testosterone replacement was shown to exert cardioprotective effects in orchiectomized rats (48). In another study in isolated rat hearts exposed to acute ischemia, testosterone significantly reduced norepinephrine release and consequent arrhythmias (49).

### Arrhythmogenic Cardiomyopathy

Arrhythmogenic right ventricular cardiomyopathy (ARVC) is an inherited heart disease that is associated with life-threatening ventricular arrhythmias. It is one of the leading causes of SCD, especially in the young, athletic population (50). While risk stratification traditionally relied on measurements of structural dysfunction and electrophysiological indices (51), there have been recent attempts to identify circulating biomarkers for prediction of arrhythmic risk. Among these, sex hormones, and particularly testosterone, seem to play an important role in arrhythmogenesis. In the study by Akdis et al. (52) our group investigated the role of sex hormones and their association with major arrhythmic cardiac events (MACE) in patients with ARVC. We observed that elevated serum testosterone levels were independently associated with MACE in male ARVC patients, whereas estradiol levels were significantly lower in female patients with MACE. These findings have recently been validated in another study by Ren et al. (53) in a different ARVC cohort. The study showed that testosterone levels were a strong predictor of future adverse arrhythmic events in male patients with ARVC, independently from baseline systolic function. However, there was no association between the circulating levels of sex hormones and future heart failure events.

It is important to note that SCD may occur during early, clinically occult stage of ARVC, which still bears the pathological hallmarks of progressive cardiomyocyte loss and fibrofatty infiltration. Hence, there is an underlying electrophysiological substrate in ARVC that can facilitate reentry. This is in contrast with the mechanisms of arrhythmogenesis in patients with outflow tract ventricular tachyarrhythmias in the absence of structural heart disease. The study by Hu et al. (54) did not reveal any differences in circulating testosterone levels between diseased and control males with such idiopathic arrhythmias, while estradiol levels were indeed lower in diseased males. While the proarrhythmogenic effect of testosterone in ARVC may be partially related to increased apoptosis and lipogenesis, as shown in the study by Akdis et al. (52) arrhythmia susceptibility in patients with idiopathic outflow tract tachyarrhythmias may be due to estrogen deficiency and its consequences on the cardiac calcium and potassium channels, as discussed previously.

### Takotsubo Syndrome

Takotsubo Syndrome is characterized by acute and mostly transient left ventricular dysfunction, which is often preceded by emotional or physical triggers. The patients have a substantial risk for MACE (55). The fact that this condition typically occurs in post-menopausal women (up to 90% of cases) has pointed out the role of estrogen deficiency as a causal factor, although pathophysiological mechanisms underlying the disease are still largely debated. The lack of estrogen together with an excess in catecholamines may not only underlie the pathophysiology of the disease itself, but also the risk of arrhythmic complications. This has been shown in a small study by El-Battrawy et al. (56) on hiPSC-CMs, which demonstrated that catecholamine excess may increase reactive oxygen species (ROS) production, which in turn enhances the late sodium current (I_Na_) and suppresses the I_to_, causing prolongation of AP duration. Interestingly, when these hiPSC-CMs were treated with estradiol, this reduced their sensitivity to catecholamines by reducing adrenoreceptor expression, and thus, showed a protective effect of estrogen in the context of this disease. Further studies are needed to determine the exact pathophysiologic and prognostic role of sex hormones in Takotsubo Syndrome.

### Cardiac Channelopathies

#### Long QT Syndrome (LQTS)

This condition defines prolonged rate-corrected QT intervals (QTc) in patients who are at risk for ventricular tachyarrhythmias (typically TdP) and SCD (36). LQTS can be either congenital or acquired (as is the case for drug-induced LQTS). Female sex is known to be an independent risk factor, as females have 10–20 ms longer QTc intervals. However, the gender difference only manifests after puberty, suggesting that female sex hormones may play a role in the increased propensity for arrhythmias (36, 57, 58).

The QTc is most affected by alterations in phase 2 and 3 of the AP, which are dominated, respectively, by the L-type calcium current (upregulation lengthens the QTc) and the delayed rectifier potassium currents (I_kr_ and I_ks_ upregulation shortens the QTc). As mentioned previously, estrogen, progesterone and testosterone have varying effects on these currents, which could explain the gender differences (7). While the hypothesis that increased estrogen might be responsible for arrhythmogenesis in females has not been proven in humans yet, there are several clinical studies showing that QTc and arrhythmogenicity may increase in in males with decreased testosterone. A recent study by Salem et al. (59) has reported that male hypogonadism secondary to androgen-deprivation therapies (ADTs) is associated with acquired LQTS and increased incidence of TdP. Furthermore, the same group has also studied hiPSCs from men and treated them either with ADTs and dihydrotestosterone, reporting that while the ADTs indeed prolonged the QT interval, this was acutely reversed upon dihydrotestosterone administration (60).

#### Brugada Syndrome

This genetically-determined disease is characterized by the appearance of a coved-type ST segment elevation in the right precordial ECG leads, and puts the patients at significant risk for SCD in the absence of an underlying structural heart disease (61). The most common genetic mutations found in affected patients (up to 25%) are loss-of-function mutations in the SCN5A gene, which encodes the α subunit of the cardiac sodium channel protein (Nav_1.5_) (62). Despite the autosomal dominant inheritance of the disease, its incidence is significantly higher in males (8:1 in Western countries and 9:1 in Asia). Indeed, a study by Shimizu et al. (42) showed that males with the Brugada Syndrome had significantly higher testosterone levels as compared to their control counterparts, even after adjusting for age, exercise, stress, smoking habits, and medications. Furthermore, there are a few interesting case reports correlating the Brugada ECG pattern to blood testosterone levels. Matsuo et al. (63) described two cases of males with an asymptomatic Brugada ECG pattern, which disappeared following surgical castration for prostate cancer. Another recently published case study by Sichrovsky et al. (64) has demonstrated the development of a previously unrecognized Brugada pattern and SCD in a genetic female living as a transgender male through the use of exogenous testosterone.

As previous literature has suggested, the accentuation of the epicardial AP notch and loss of its dome induced by a potassium channel opener, seems to be the cause of the typical Brugada-type ST segment elevation. This can result in phase II reentry and trigger ventricular tachycardia or ventricular fibrillation (65). Moreover, it matches the evidence of the sodium channel being the primary gene candidate for the Brugada Syndrome. Since either a decrease in the density or an acceleration of inactivation of the sodium channel would leave I_to_ unopposed during the early phases of the AP, the mechanism of the AP notch accentuation may indeed be due to a more prominent I_to_, which is physiologically more expressed in males as compared to females. This has been proven in a dog model, showing that the I_to_ current density of the RV epicardium is significantly higher in males than in females, culminating in increased transmural dispersion of repolarization (4). This substrate facilitates the presence of the Brugada-type ECG pattern and occurrence of arrhythmias in males. Furthermore, as previously mentioned, testosterone may augment outward repolarizing currents (such as I_kr_ and I_ks_), and thus, lead to loss of the AP dome, explaining the male predominance of the Brugada Syndrome. A recently published *in vitro* study by Yang et al. (66) examining the effects of estrogen and testosterone on the wild-type and the mutant Nav_1.5_ has shown no influence of these hormones on either channel, but this study had the limitation of lacking an *in vivo* validation.

#### Catecholaminergic Polymorphic Ventricular Tachycardia

CPVT is an inherited arrhythmic syndrome, which affects 1 in 10,000 individuals. It is characterized by potentially lethal ventricular arrhythmias, which are often biventricular or polymorphic in origin, and which mostly appear following an adrenergic challenge precipitated by emotional or physical stress (67). CPVT arises from disrupted intracellular calcium homeostasis and is most often associated with gene abnormalities involving the RyR2. The resulting dysregulated RyR2-mediated leak of sarcoplasmic reticular calcium elevates cytosolic calcium, increasing electrogenic sodium/calcium exchanger and/or calcium-activated chloride transient inward currents. The consequently occurring delayed afterdepolarizations may trigger arrhythmic events. Interestingly, the majority of phenotypes related to *RyR2* mutations show a higher mortality in males as compared to females. The molecular basis for this entity has been explored in a recent study by Saadeh et al. (68) in a murine model of CPVT (homozygotic RyR2^S/S^), in which they investigated the impact of gender on the expression levels of molecular determinants of calcium homeostasis and conduction velocity. While the authors have found no difference in the expression levels of calcium homeostasis proteins, they showed a decreased Cx43 expression, which correlated with slowed conduction velocity in female mice, but not in males.

## Hormone Replacement Therapy and Arrhythmias

Male hypogonadism, which may arise from multiple etiologies including androgen-deprivation therapy (ADT), has been reported as a risk factor for acquired LQTS and the occurrence of TdP. This has led to multiple pharmacovigilance studies assessing the link between hormone replacement therapy (HRT) and the incidence of ventricular arrhythmias. Interestingly, this has helped to shed some light on the link between arrhythmias and sex hormones, supporting the hypothesis that hypogonadism is a correctable and identifiable risk factor for TdP, especially in men. This has clinical implications, for example while considering ADT, which is the cornerstone of the treatment of prostate cancer.

### Ventricular Arrhythmias

Male hypogonadism is a condition in which clinical symptoms occur due to testosterone deficiency (69). As discussed previously, low testosterone values may be associated with a higher risk of ventricular arrhythmias and SCD due to a lengthening of the myocardial repolarization phase. In fact, the QTc values of males after puberty are significantly shorter and aging men with decreasing testosterone levels seem to have a gradual increase in QTc. Some types of male hypogonadism can be treated with testosterone replacement therapy (TRT). The data available on the effects of TRT on cardiovascular risks are contradicting. The RHYME study, which investigated hypogonadal men receiving TRT, did not find any increased cardiovascular risk (70). The same results were shown in a systematic review by Corona et al. (71) which reported the absence of a causal role between TRT and cardiovascular events. Interestingly, a study by Muensterman et al. (58) reported that in older men, the use of transdermal testosterone combined with oral progesterone attenuates drug-induced QTc lengthening. This shows that there may be a probable protective role of both testosterone and progesterone on arrhythmia occurrence due to prolonged QTc.

Concerning hormone replacement in females, there are several studies in post-menopausal women showing that estrogen replacement therapy (ERT) prolongs the QTc interval. Indeed, as mentioned above, estrogen has a lengthening effect on the myocardial repolarization phase. This effect is not evident in female children, but only manifests itself after adolescence, and significantly decreases after menopause (72). A study on hormone replacement for 1 year confirmed that the use of ERT increases the QTc interval (73). Interestingly, this effect was not seen in combined estrogen-progestin replacement therapies, strengthening the theory that progesterone most likely has a similar effect on the QTc interval as testosterone. It is important to note that a study by Saba et al. (74) did not find any significant difference in QTc interval between premenopausal, post-menopausal and post-menopausal women treated with hormone replacement. A major limitation of this study was that it was not clear whether hormone replacement consisted of estrogen alone or combined with progesterone in the study population (74).

## Conclusions

Gender is known to be an independent risk factor for some types of cardiac arrhythmias. However, the link between the sex hormones and susceptibility to arrhythmias is still a matter of debate. Nonetheless, despite conflicting results, these hormones may influence arrhythmia occurrence both in the presence or absence of underlying structural heart disease. Further studies, which validate or contradict the already present literature, will be of invaluable importance to fully understand the pathophysiological mechanisms that lie at the basis of arrhythmogenesis.

## Author Contributions

SC manuscript drafting and data collection. AS manuscript drafting and review. AG and DA data collection and manuscript review. CB funding recruitment and manuscript review. FD coordinator, manuscript structuring, and manuscript review. All authors contributed to the article and approved the submitted version.

## Conflict of Interest

The authors declare that the research was conducted in the absence of any commercial or financial relationships that could be construed as a potential conflict of interest.
